# *Amauroderma rugosum* Protects PC12 Cells against 6-OHDA-Induced Neurotoxicity through Antioxidant and Antiapoptotic Effects

**DOI:** 10.1155/2021/6683270

**Published:** 2021-02-03

**Authors:** Jingjing Li, Renkai Li, Xiaoping Wu, Ruby Lai-Chong Hoo, Simon Ming-Yuen Lee, Timothy Man-Yau Cheung, Bryan Siu-Yin Ho, George Pak-Heng Leung

**Affiliations:** ^1^Department of Pharmacology and Pharmacy, The University of Hong Kong, Hong Kong, China; ^2^State Key Laboratory of Quality Research in Chinese Medicine and Institute of Chinese Medical Sciences, University of Macau, Macao, China; ^3^Tian Ran Healthcare Limited, Hong Kong, China

## Abstract

*Amauroderma rugosum* (AR) is a dietary mushroom in the *Ganodermataceae* family whose pharmacological activity and medicinal value have rarely been reported. In this study, the antioxidant capacity and neuroprotective effects of AR were investigated. The aqueous extract of AR was confirmed to contain phenolic compounds, polysaccharides, and triterpenes. The results of 2,2-diphenyl-1-picryl-hydrazyl-hydrate (DPPH) and total antioxidant capacity assays revealed that AR extract scavenged reactive oxygen species. Moreover, AR extract decreased the cytotoxicity, oxidative stress, mitochondrial dysfunction, and apoptosis of PC12 cells induced by 6-hydroxydopamine (6-OHDA). In addition, 6-OHDA upregulated the expressions of proapoptotic proteins and downregulated the Akt (protein kinase B)/mTOR- (mammalian target of rapamycin-) and MEK (mitogen-activated protein kinase kinase)/ERK- (extracellular signal-regulated kinases-) dependent signaling pathways. These effects of 6-OHDA were abolished or partially reversed by AR extract. Furthermore, the neuroprotective effects of AR in 6-OHDA-treated PC12 cells were significantly abolished by Akt and MEK inhibitor. Thus, AR extract possesses neuroprotective effects, probably through its antioxidant and antiapoptotic effects. These findings suggest the potential application of AR in the prevention or treatment of oxidative stress-related neurodegenerative diseases such as Parkinson's disease.

## 1. Introduction

To date, Parkinson's disease remains the second most common neurodegenerative disease worldwide, and its incidence is increasing in people over 60 years of age [1]. It is characterized by a selective and progressive loss of dopaminergic neurons in the substantia nigra pars compacta, leading to serious movement disturbances including postural instability, uncontrollable tremors, rigidity, and bradykinesia [2]. Currently, available treatments for Parkinson's disease include dopaminergic replacement therapy and deep brain stimulation therapy [3]. However, neither of these treatments can halt nor slow the progression of Parkinson's disease. Therefore, it is crucial to develop novel drugs that can slow the neurodegenerative process.

Although the pathological mechanisms of Parkinson's disease remain elusive, accumulating scientific evidence suggests that oxidative stress-induced cell injury plays an indispensable role in the degeneration of dopaminergic neurons [4]. Interrupting the physiological maintenance of redox potential severely interferes with many biological processes in neurons, eventually leading to cell apoptosis [5]. Oxidative stress occurs when the rate of reactive oxygen species (ROS) scavenging is overwhelmed by the rate of ROS production [6]. Excessive accumulation of ROS in dopaminergic neurons can damage most biological molecules, including lipids, proteins, and nucleic acids, thereby activating the intracellular inflammatory response, which induces cellular damage, mitochondrial dysfunction, oxidative DNA injury, and neuroinflammation [7–9]. Therefore, decreasing oxidative injury in dopaminergic neurons has been widely proposed as an effective approach for the treatment of Parkinson's disease.

Various experimental models have been established for investigating the role of oxidative stress in dopaminergic neuronal degeneration. These models involve the use of neurotoxins such as 1-methyl-4-phenyl-1,2,3,6-tetrahydropyridine (MPTP), rotenone, 1,1′-dimethyl-4,4′-bipyridinium dichloride (paraquat) and 6-hydroxydopamine (6-OHDA) [5]. The compound 6-OHDA, the hydroxylated analog of natural neurotransmitter dopamine, is a widely used neurotoxin that can be applied to establish different *in vitro* and *in vivo* Parkinson disease models. It is taken up by dopaminergic neurons via dopamine and norepinephrine transporters and is subsequently oxidized intracellularly, thereby releasing ROS including hydrogen peroxide, superoxide, and hydroxyl radicals [10–12].

*Amauroderma rugosum* (AR) is a basidiomycete in the *Ganodermataceae* family. This mushroom has a black stipe and a white surface covered with numerous pores. A notable characteristic of the mushroom is that its surface becomes red when it is scratched. Hence, it is also known as “blood Linzhi” in Chinese. Although AR is commonly consumed by people in China and South Asia, very few scientific studies have explored its beneficial effects on health or its medicinal value. Nevertheless, a previous study reported that the extract of AR mycelia has antioxidant and anti-inflammatory effects in lipopolysaccharide-stimulated RAW 264.7 cells. Thus, AR might be a potential therapeutic agent or health supplement useful in the management of oxidative stress-related diseases [13]. The aims of this study were to investigate the antioxidant capacity and neuroprotective activity of AR in a 6-OHDA-induced neurodegenerative cell model and to elucidate its underlying mechanisms of action.

## 2. Materials and Methods

### 2.1. Chemicals and Reagents

Dulbecco's modified Eagle's medium (DMEM), fetal bovine serum (FBS), 4′,6-diamidino-2-phenylindole (DAPI), penicillin–streptomycin, and 0.25% (*w*/*v*) trypsin containing 1 mM ethylenediaminetetraacetic acid were purchased from Invitrogen (Carlsbad, CA), and 6-OHDA, 2,2-diphenyl-1-picryl-hydrazyl-hydrate (DPPH), vitamin C, dimethyl sulfoxide (DMSO), Akt inhibitor IV, MEK inhibitor (PD 98059), and 3-(4,5-dimethylthiazol-2-yl)-2,5-diphenyltetrazoliumbromide (MTT) were purchased from Sigma-Aldrich (St. Louis, MO). Nerve growth factor (NGF) was obtained from R&D Systems (Minneapolis, MN, USA). A total antioxidant capacity assay kit was purchased from Abcam (Cambridge, UK). A lactate dehydrogenase (LDH) cytotoxicity assay kit was purchased from Cayman Chemical (Ann Arbor, MI). A Caspase 3/7 activity detection kit was obtained from Promega (Madison, USA). Antibodies for western blotting were purchased from Cell Signaling Technology (Danvers, MA). All chemicals were dissolved in appropriate solvents and stored at −20°C before use to maintain their chemical stability.

### 2.2. Reflux Extraction of AR

Fruiting bodies of AR were provided by Hong Kong Ganoderma Centre Limited (Hong Kong, China), an organic farm that had been granted an organic crop production certificate by the Hong Kong Organic Resource Centre. The samples were dried in an oven and ground into powder. A reflux system for the extraction process was used to prepare the crude extract. Two grams of the powdered sample was extracted with 50 mL of distilled water at 95 ± 2°C for 60 min. The crude extract was centrifuged at 4000 rpm for 20 min. Afterward, the supernatant was collected, and the sample residue was reextracted twice via the steps described above. Subsequently, all extracts were pooled, filtered, and concentrated to 80 mL with a rotary evaporator. The extract was stored at -20°C until further use.

### 2.3. Determination of Total Phenolic Compounds, Polysaccharides, and Triterpenes

To measure the total phenolic content of AR extract, 50 *μ*L of 10% Folin-Ciocalteu phenol reagent was added to 50 *μ*L AR extract and incubated in dark at room temperature for 3 min. Afterwards, 100 *μ*L of 10% Na_2_CO_3_ was added to the mixture for 1 h. The absorbance at 750 nm was measured with a microplate absorbance reader. Gallic acid was used as a standard phenolic compound. All determinations were expressed as mg gallic acid equivalent per g (mg GAE/g).

Before the measurement of the total polysaccharides, 0.1 mL AR extract was precipitated with 1 mL of 95% ethanol overnight at 4°C. The precipitate was collected by centrifugation at 10,000 rpm for 10 min at 4°C. Then, the precipitate was dissolved in 50 *μ*L water. Total polysaccharide content was measured by adding 2.5 *μ*L phenol (80%) and then 125 *μ*L concentrated sulfuric acid. After incubation for 10 min, the mixture was shaken and then incubated at 30°C for 20 min. The absorbance at 490 nm was measured with a microplate absorbance reader using glucose as standard. The results were expressed as mg glucose equivalent per g (mg GE/g).

To measure the total triterpenes, 100 *μ*L AR extract was transferred to 15 mL tube and evaporated to dryness using nitrogen flow. Then, 0.4 mL 5% vanillin–acetic acid solution and 1 mL perchloric acid were added into the tube, mixed and incubated at 60°C for 15 min. Afterwards, 5 mL acetic acid was added and incubated at room temperature for 15 min. The absorbance at 549 nm was measured with a microplate absorbance reader. A solution of oleanolic acid was used as the standard. The results were expressed as mg oleanolic acid equivalent per g (mg OA/g).

### 2.4. DPPH Assay

The free radical scavenging capacity (SC) of AR extract was measured with DPPH assays. Briefly, 5 *μ*L of AR extract was mixed with 195 *μ*L of DPPH solution (24 mg/L) in a 96-well plate. The reaction proceeded in the dark for 60 min. Afterward, the absorbance of the reaction mixture at 515 nm was measured with a microplate absorbance reader. Vitamin C dissolved in distilled water served as the positive control. The SC_50_ was estimated as the concentration of extract that scavenged 50% of the free radicals.

### 2.5. Total Antioxidant Capacity Assay

The total antioxidant capacity (TAC) of AR extract was evaluated with a TAC Assay Kit, which measured the ability of antioxidants to reduce Cu^2+^ to Cu^+^. The resulting Cu^+^ formed a colored complex with a specific dye reagent in the assay kit. In brief, 5 *μ*L of AR extract was diluted into 100 *μ*L with deionized water and then mixed with 100 *μ*L of Trolox standard in a 96-well plate. The reaction was performed by the addition of 100 *μ*L of Cu^2+^ working solution, and the plate was shaken for 90 min in dark at room temperature. The absorbance at 570 nm was measured with a microplate absorbance reader.

### 2.6. Cell Culture and Treatment

PC12 rat pheochromocytoma cells were obtained from the American Type Culture Collection (Manassas, VA). The cells were cultured in DMEM supplemented with 10% heat-inactivated FBS and 1% penicillin-streptomycin and then incubated at 37°C in a humidified atmosphere with 5% CO_2_. For the experiments with 6-OHDA, PC12 cells in DMEM with low serum (0.5% FBS) were seeded in 12- or 96-well plates. The cells were incubated with different concentrations of AR extract (0-2 mg/mL) for 2 h and then treated with 500 *μ*M 6-OHDA for 24 h. The differentiation of PC12 cells was described previously [14]. Neuronal differentiation was induced in the culture medium by treating the PC12 cells with 100 ng/mL NGF and 1% FBS for 5 days. Differentiated PC12 cells were then collected for further analysis and compared with nondifferentiated treated PC12 cells.

### 2.7. Cell Viability Assay

The cell viability was measured with MTT assays according to the manufacturer's protocol. In brief, the cultured medium was discarded, and the cells were incubated with MTT solution (at a final concentration of 0.5 mg/mL) for 4 h at 37°C. Dimethyl sulfoxide was then added to lyse the cells and dissolve the violet formazan crystals that had formed inside the cells. The absorbance at 570 nm was measured with a microplate absorbance reader.

### 2.8. LDH Assay

Cellular injury was determined by measurement of the LDH released into the culture medium. LDH activity was measured with a detection kit according to the manufacturer's instructions. The absorbance at 490 nm was measured with a microplate absorbance reader.

### 2.9. DAPI and Annexin V-Fluorescein Isothiocyanate (FITC)/Propidium Iodide (PI) Staining

After drug treatment, the PC12 cells were washed twice with cold PB and stained with DAPI (2.0 *μ*g/mL) for 20 min. The images were then captured by fluorescence microscopy (IN CELL Analyzer, GE Healthcare Life Sciences, USA). The PC12 cells were also resuspended in binding buffer and then stained with annexin V-FITC and PI (1.0 mg/mL) for 20 min. The stained cells were analyzed immediately with a flow cytometer (BD Biosciences, USA). Ten thousand events were counted for each sample. The data were analyzed in the FlowJo software (BD Biosciences, USA).

### 2.10. Mitochondrial Membrane Potential

The PC12 cells were incubated with JC-1 dye (3 *μ*g/mL) for 20 min. A portion of the cells was photographed by a fluorescence microscopy, and the remaining cells were washed twice with warm PBS and examined by flow cytometry. The intensity of red fluorescence (excitation 560 nm, emission 595 nm) and green fluorescence (excitation 485 nm, emission 535 nm) was determined in ImageJ (National Institutes of Health, USA). The ratio of red/green fluorescence was calculated for semiquantitative assessment of mitochondrial polarization states.

### 2.11. Analysis of Mitochondrial Respiration

Mitochondrial oxygen consumption rate (OCR) was measured using Seahorse XFe24 Analyzer (Seahorse Biosciences, MA, USA). PC12 cells (8 × 10^3^ cells/well) were seeded into a Seahorse XF 24 well culture microplates and incubated overnight at 37°C in a humidified atmosphere with 5% CO_2_. After drug treatment, the cultural medium was replaced with Seahorse base medium and incubated in a non-CO_2_ incubator for 1 h. PC12 cells were sequentially treated with 1 *μ*M oligomycin (Oligo), 1 *μ*M carbonyl cyanide-4-(trifluoromethoxy) phenylhydrazone (FCCP), and 1 *μ*M rotenone plus 1 *μ*M antimycin A (R + A). OCR was calculated using the Seahorse software. After finishing the assay, the cells were lysed with RIPA buffer (200 *μ*L/well), and the protein concentration was measured by bicinchoninic acid assay. OCR was normalized to the protein content and presented as pmol/min/*μ*g protein.

### 2.12. Detection of ROS

ROS was detected with CM-H_2_DCFDA staining. A portion of the cells was photographed under fluorescence microscopy, and the remaining cells were examined by flow cytometry. The intensity of green fluorescence (excitation 485 nm, emission 535 nm) was determined in the ImageJ software.

### 2.13. Caspase 3/7 Activity Assay

Caspase 3/7 activity in PC12 cells was evaluated with a caspase 3/7 activity detection kit according to the manufacturer's protocol. Luminescence signals were recorded with a microplate absorbance reader.

### 2.14. Western Blot Analysis

Protein was extracted from PC12 cells with lysis buffer containing 1% phenylmethylsulfonyl fluoride and 1% protease inhibitor. Lysates were centrifuged at 12,500 × g for 20 min at 4°C, and the supernatant was collected. The total protein concentration was determined with bicinchoninic acid assays. Equal amounts of protein were subjected to sodium dodecyl sulfate-polyacrylamide gel electrophoresis and then electrically transferred onto a polyvinylidene difluoride membrane, which was subsequently blocked with 5% nonfat milk in Tris-buffered saline containing 0.1% Tween-20 for 1 h. The membrane was subsequently incubated with primary antibodies against mTOR, phospho-mTOR (Ser2448), Akt, phospho-Akt (Ser473), ERK1/2, phospho-ERK1/2 (Thr202/Tyr204), MEK, phospho-MEK (Ser217/221), cleaved-PARP (Asp214), cleaved-caspase 3 (Asp175), cleaved-caspase 9 (Asp315), or GAPDH overnight at 4°C. After being washed with PBS, the membrane was incubated with horseradish peroxidase-conjugated secondary antibodies for 1 h at room temperature. After repeated washes with PBS, proteins were visualized by enhanced chemiluminescence. Images of protein bands were captured, and densitometric measurements of band intensity were performed with a ChemiDoc XRS Molecular Imager (Bio-Rad Laboratories, Hercules, CA, USA).

### 2.15. Data and Statistical Analysis

Data are expressed as the mean ± standard deviation (SD) of at least three independent experiments. Statistical analyses were performed with one-way ANOVA followed by Tukey's multiple comparison test (two or more groups) in the GraphPad Prism 6.0 software (GraphPad Software Inc., San Diego, CA, USA). *p* < 0.05 was considered statistically significant.

## 3. Results

### 3.1. Chemical Contents of AR Extract

Major contents of AR extract including total phenolic compounds, polysaccharides, and triterpenes were measured by chemical assays. The content of total phenolic compounds of AR extract was 5.53 ± 0.11 mg GAE/g of dry weight. The content of total polysaccharides in AR extract was 1.12 ± 0.23 mg GE/g of dry weight. The content of total triterpenes was 3.20 ± 0.14 mg OA/g of dry weight.

### 3.2. Antioxidant Capacity of AR Extract in DPPH and TAC Assays

**Figure 1:**
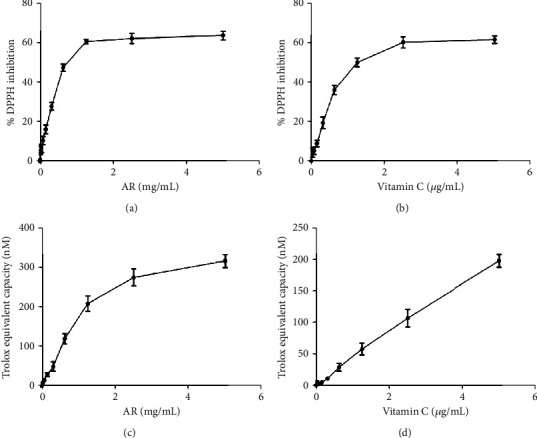
Antioxidant capacity of AR extract and vitamin C. The effects of different concentrations of (a) AR extract and (b) vitamin C on scavenging DPPH were studied. The antioxidant capacity is expressed as percentage DPPH inhibition. The effects of different concentrations of (c) AR extract or (d) vitamin C on total antioxidant capacity were also studied by measuring their ability to reduce Cu^2+^ to Cu^+^. The antioxidant effect is expressed as Trolox equivalent antioxidant capacity. Values are means ± SD of three independent experiments.

The antioxidant capacity of AR extract was studied with DPPH and TAC assays. The ability of AR extract and vitamin C (which served as a positive control) to scavenge DPPH• free radicals increased with the tested concentration (Figures 1(a) and 1(b)). The SC_50_ of AR extract and vitamin C in DPPH assays was 0.58 mg/mL and 1.34 *μ*g/mL, respectively. Similarly, AR extract and vitamin C reduced Cu^2+^ radicals in TAC assays in a concentration-dependent manner (Figures 1(c) and 1(d)).

### 3.3. Neuroprotective Effect of AR Extract in PC12 Cells

**Figure 2:**
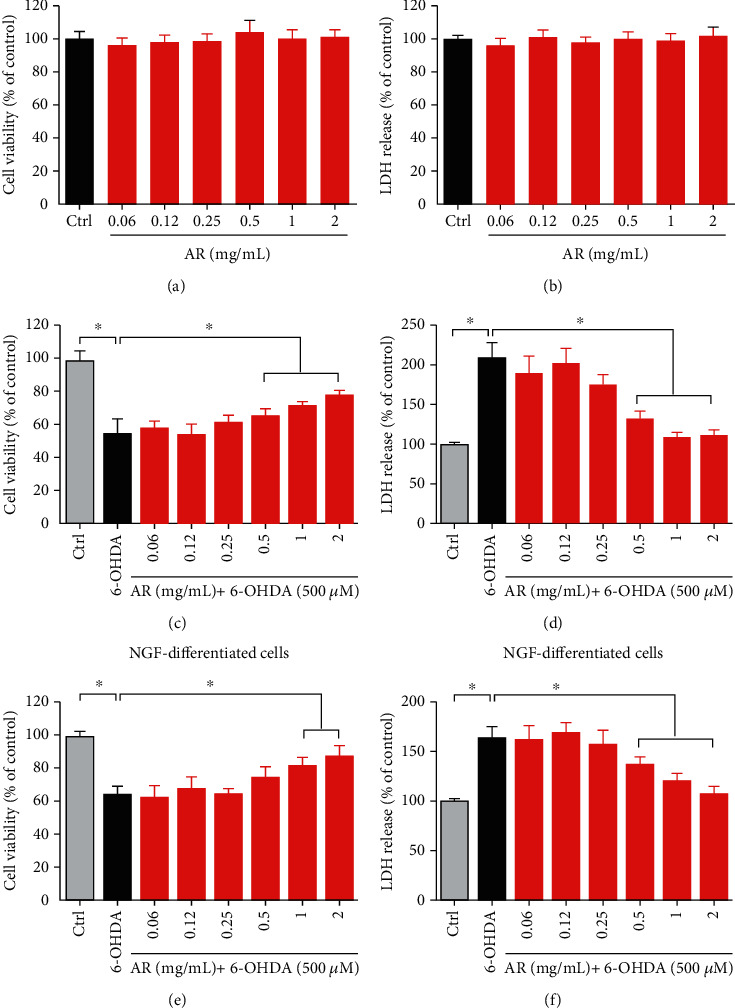
AR extract protects PC12 cells against 6-OHDA-induced cell cytotoxicity. PC12 cells were treated with various concentrations of AR extract (0.06–2 mg/mL) or vehicle (control) for 24 h. Then, (a) cell viability and (b) LDH release were examined with MTT and LDH assays, respectively. PC12 cells were then incubated with different concentrations of AR extract (0–2 mg/mL) for 2 h and subsequently treated with 500 *μ*M 6-OHDA for 24 h. Cells without the treatment with AR extract and 6-OHDA served as controls. (c) Cell viability and (d) LDH release were again studied. (e, f) NGF-differentiated PC12 cells were treated with AR extract (0–2 mg/mL) for 2 h prior to treatment with 500 *μ*M 6-OHDA for 24 h. Then, (e) cell viability and (f) LDH release were examined with MTT and LDH assays, respectively. Data are presented as a percentage of control group values (mean ± SD of three independent experiments). ^∗^*p* < 0.05 indicates a statistically significant difference.

PC12 cells were used as the cell model to study the neuroprotective effect of AR extract. First, the cytotoxic effect of AR extract itself was evaluated by incubation of PC12 cells with various concentrations of AR extract (0.06–2 mg/mL) for 24 h. The MTT assays showed that AR extract did not affect the viability of PC12 cells in the concentration range of 0.06 to 2 mg/mL (Figure 2(a)). In addition, LDH release by PC12 cells was not affected by AR extract in the same concentration range (Figure 2(b)). Therefore, this concentration range of AR extract was applied in subsequent experiments. Under 6-OHDA (500 *μ*M) treatment, the viability of PC12 cells decreased by 45%, and LDH release increased by 110% (Figures 2(c) and 2(d)). AR extract protected PC12 cells against 6-OHDA-induced cell death and LDH release, in a concentration-dependent manner. We further investigated the protective effects of AR in NGF-differentiated PC12 cells. Consistent with data of nondifferentiated PC12 cells, AR significantly increased the cell viability and reduced LDH release in NGF-differentiated PC12 cells (Figures 2(e) and 2(f)).

### 3.4. Antioxidant Activity of AR Extract in PC12 Cells

**Figure 3:**
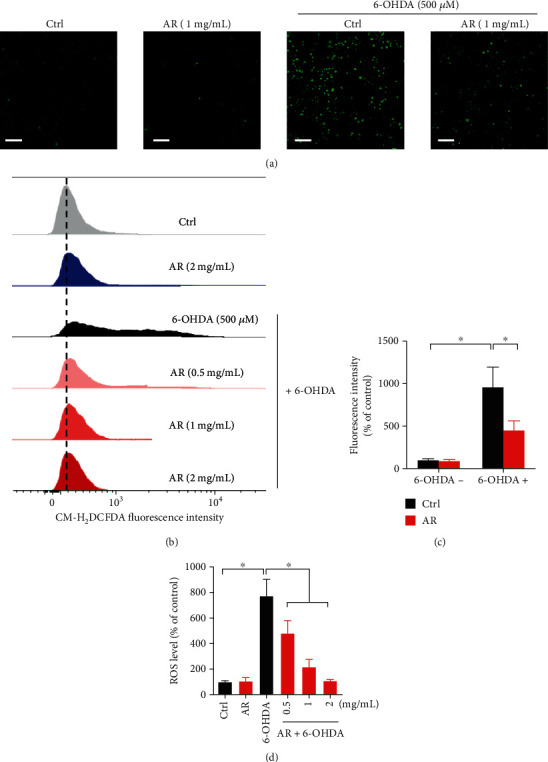
AR extract decreases 6-OHDA-induced ROS generation in PC12 cells. PC12 cells were pretreated with different concentrations of AR extract (0.5–2 mg/mL) or vehicle for 2 h and then treated with or without 500 *μ*M 6-OHDA for 4 h. Cells without treatment with AR extract and 6-OHDA served as the control. (a) ROS generation in PC12 cells was detected by CM-H2DCFDA staining. ROS in the cells was indicated by the green signals. Scale bar: 200 *μ*m. (b) Flow cytometry analysis of ROS levels in PC12 cells after CM-H2DCFDA staining. ROS levels in (c) microscopy images and (d) flow cytometry were quantified. Data are presented as a percentage of control group values (mean ± SD of three independent experiments). ^∗^*p* < 0.05 indicates a statistically significant difference.

The *in vitro* antioxidant activity of AR extract was studied in PC12 cells treated with 6-OHDA. The intracellular ROS generation was reflected by the green florescent signal produced by the probe CM-H2DCFDA. AR extract itself had no effect on ROS generation in PC12 cells (Figures 3(a) and 3(c)). The ROS level in PC12 cells was elevated significantly, by 9.6-fold, after treatment with 6-OHDA, but the increase was only 4.6-fold when the cells were pretreated with 1 mg/mL AR extract. Similar results were observed in flow cytometry analysis. The 6-OHDA-induced ROS production in PC12 cells was inhibited by AR extract in a concentration-dependent manner (Figures 3(b) and 3(d)).

### 3.5. Mitochondrial Protective Effect of AR Extract on PC12 Cells

**Figure 4:**
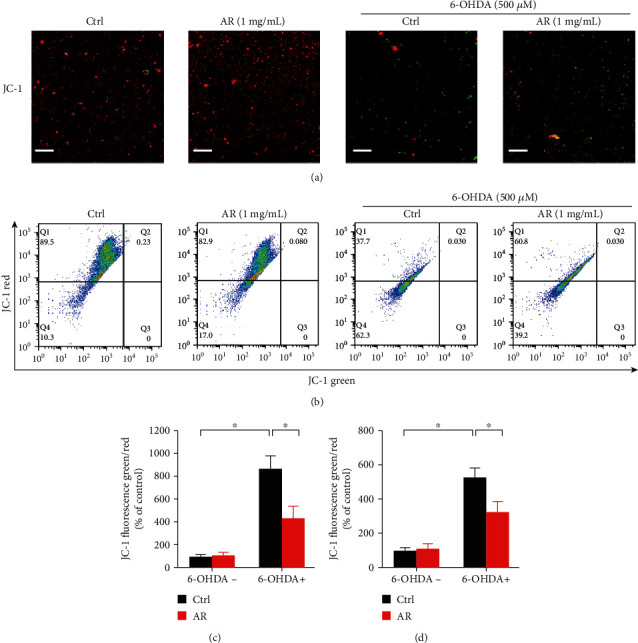
AR extract ameliorates 6-OHDA-induced loss of mitochondrial membrane potential in PC12 cells. PC12 cells were pretreated with 1 mg/mL AR extract or vehicle for 2 h and then treated with or without 500 *μ*M 6-OHDA for 24 h. (a) Mitochondrial membrane potential in PC12 cells was detected with JC-1 staining. Red and green fluorescence signals indicated JC-1 aggregates and monomers, respectively. Scale bar: 200 *μ*m. (b) Flow cytometry analysis of mitochondrial membrane potential in PC12 cells after JC-1 staining. The mitochondrial membrane potential in (c) microscopy image and (d) flow cytometry was quantified. Data are presented as a percentage of control group values (mean ± SD of three independent experiments). ^∗^*p* < 0.05 indicates a statistically significant difference.

Mitochondrial membrane potential is an important indicator of mitochondrial function, and the loss of mitochondrial membrane potential is typically regarded as a hallmark of apoptosis [15]. The membrane-permeant JC-1 dye was used for evaluating the mitochondrial membrane potential. The color of JC-1 dye changes from red to green when the mitochondrial membrane potential decreases. Green fluorescence signals were scarcely observed in both the control and AR extract-treated PC12 cells. However, the ratio of the green fluorescence to red fluorescence signal significantly increased, by 8.7-fold, under 500 *μ*M 6-OHDA treatment (Figures 4(a) and 4(c)), revealing disruption of the mitochondrial membrane potential. However, the 6-OHDA-induced increase in the ratio of the green fluorescence to red fluorescence signal was only 4.3-fold when the PC12 cells were pretreated with 1 mg/mL AR extract. Similar results were observed with flow cytometry. The ratio of the green fluorescence to red fluorescence signal increased by 5.3-fold under 500 *μ*M 6-OHDA treatment but by only 3.2-fold when the PC12 cells were first preincubated with 1 mg/mL AR extract (Figures 4(b) and 4(d)).

### 3.6. Protective Effect of AR Extract against 6-OHDA-Induced Mitochondrial Respiratory Dysfunction PC12 Cells

**Figure 5:**
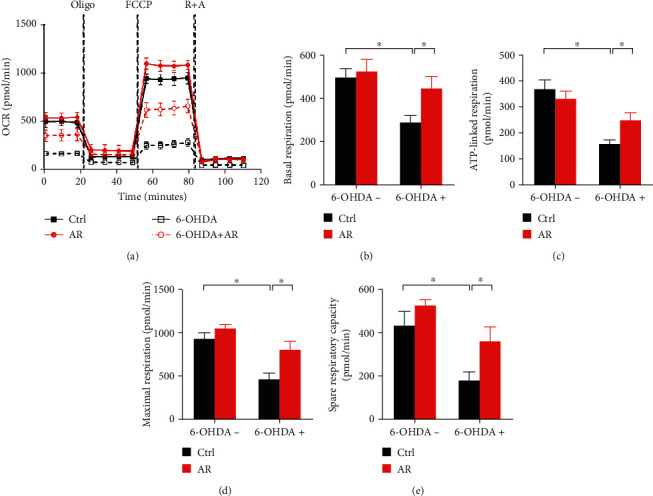
AR extract reduces 6-OHDA-induced mitochondrial respiratory dysfunction in PC12 cells. PC12 cells were pretreated with 1 mg/mL AR extract or vehicle for 2 h and then treated with or without 500 *μ*M 6-OHDA for 24 h. (a) Mitochondrial oxygen consumption rate (OCR) in PC12 cells was monitored using a Seahorse metabolic analyzer. The response of PC12 cells after addition of 1 *μ*M oligomycin (Oligo), 1 *μ*M FCCP, and 1 *μ*M rotenone plus 1 *μ*M antimycin (R + A) was recorded. (b–e) Quantitative analysis of (b) basal respiration, (c) ATP-linked respiration, (d) maximal respiration, and (e) spare respiratory capacity in PC12 cells, respectively. The values represent the mean ± SD (*n* = 3). ^∗^*p* < 0.05 indicates a statistically significant difference.

Mitochondrial respiratory function of PC12 cells was further evaluated using a Seahorse Bioscience extracellular flux analyzer. The drop of basal OCR by oligomycin could be used to deduce ATP-linked respiration. Stimulation by FCCP could result in a maximal respiration. The gap between maximal and basal OCR was the spare respiratory capacity. Addition of rotenone (mitochondrial complex I inhibitor) and antimycin (mitochondrial complex III inhibitor) into the cells could shut down the electron transfer, allowing the calculation of nonmitochondrial respiration. In comparison with control group, treatment with AR caused a mild increase in basal respiration, maximal respiration, spare respiratory capacity, and a mild decrease in ATP-linked respiration (Figure 5(a)). In contrast, 6-OHDA decreased basal respiration, ATP-linked respiration, maximal respiration, and spare respiratory capacity by 41%, 57%, 50%, and 59%, respectively (Figures 5(b)–5(e)). The 6-OHDA-induced mitochondrial respiratory dysfunction was significantly reversed by AR extract (Figure 5(a)). In comparison with 6-OHDA treatment, AR remarkably restored mitochondrial basal respiration, ATP-linked respiration, maximal respiration, and spare respiratory capacity by 31%, 25%, 37%, and 42%, respectively (Figures 5(b)–5(e)).

### 3.7. Protective Effect of AR Extract against Apoptosis in PC12 Cells

**Figure 6:**
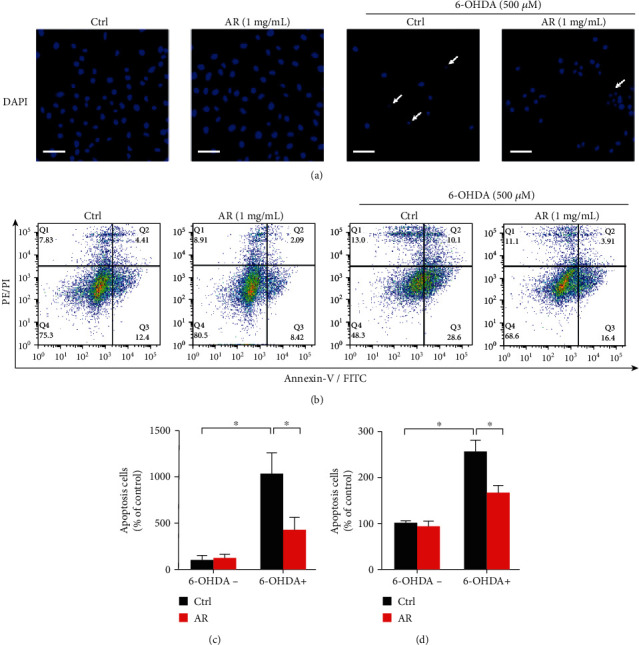
AR extract protects PC12 cells against 6-OHDA-induced apoptosis. PC12 cells were pretreated with 1 mg/mL AR extract or vehicle for 2 h and then treated with or without 500 *μ*M 6-OHDA for 24 h. (a) Apoptotic cells were identified by DAPI staining. Blue signals indicate the nuclei of PC12 cells. White arrows indicate apoptotic cells. Scale bar: 50 *μ*m. (b) Cells were double stained by annexin V and PI for 20 min and then analyzed by flow cytometry. The number of apoptotic cells in microscopy images (c) and flow cytometry (d) was quantified. Data are presented as a percentage of control group values (mean ± SD of three independent experiments). ^∗^*p* < 0.05 indicates a statistically significant difference.

Decreased mitochondrial membrane potential often leads to cell apoptosis. DAPI staining was applied to evaluate apoptosis in PC12 cells. No nuclear condensation or fragmentation was observed in the control and AR extract-treated cells. In contrast, many bright condensed dots representing apoptotic bodies were clearly identified in 6-OHDA-treated cells (Figure 6(a)). The number of apoptotic cells increased by 10.3-fold after treatment with 6-OHDA but by only 4.3-fold when the PC12 cells were first pretreated with AR extract (Figure 6(c)). Next, we further studied the antiapoptotic effect of AR extract on PC12 cells by using annexin V-FITC/PI double staining and flow cytometry (Figure 6(b)). 500 *μ*M of 6-OHDA increased the number of apoptotic cells by 2.6-fold but by only 1.7-fold when the cells were preincubated with AR extract (Figure 6(d)).

### 3.8. Inhibitory Effect of AR Extract on 6-OHDA-Induced Proapoptotic Protein Expression in PC12 Cells

**Figure 7:**
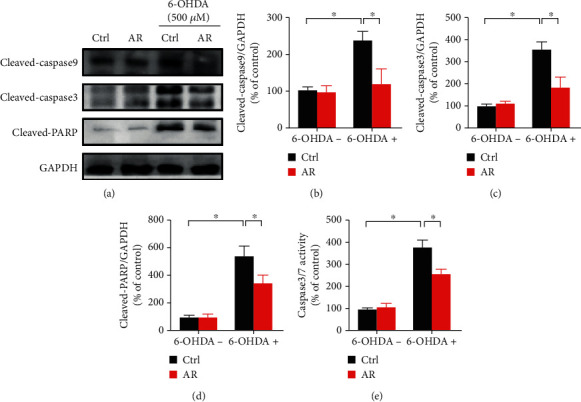
AR extract inhibits 6-OHDA-induced proapoptotic protein expression in PC12 cells. (a) PC12 cells were pretreated with 1 mg/mL AR extract or vehicle for 2 h and then treated with or without 500 *μ*M 6-OHDA for 24 h. Protein expression levels of cleaved-caspase 9, cleaved-caspase 3, and cleaved-PARP in PC12 cells were examined by Western blot analysis. (b–d) Quantitative analysis of protein expression levels. (e) Caspase 3/7 activity in PC12 cells was measured with a biochemical assay kit. Data are presented as a percentage of control group values (mean ± SD of three independent experiments). ^∗^*p* < 0.05 indicates a statistically significant difference.

Expression levels of proteins involved in apoptotic pathways were studied with Western blot analysis (Figure 7(a)). The expression levels of cleaved-caspase 9, cleaved-caspase 3, and cleaved-PARP were significantly elevated by treatment with 500 *μ*M 6-OHDA, by 137%, 255%, and 441%, respectively. The 6-OHDA-induced expression of cleaved-caspase 9 and cleaved-caspase 3 was abolished by treatment with 1 mg/mL of AR extract, whereas the 6-OHDA-induced expression of cleaved-PARP was partially inhibited by AR extract (Figures 7(b)–7(d)). Moreover, biochemical assays showed that the caspase 3/7 activity under 6-OHDA treatment increased by 3.8-fold but by only 2.6-fold when the PC12 cells were pretreated with AR extract (Figure 7(e)).

### 3.9. Restorative Effects of AR Extract on Akt/mTOR and MEK/ERK Signaling Pathways in PC12 Cells

**Figure 8:**
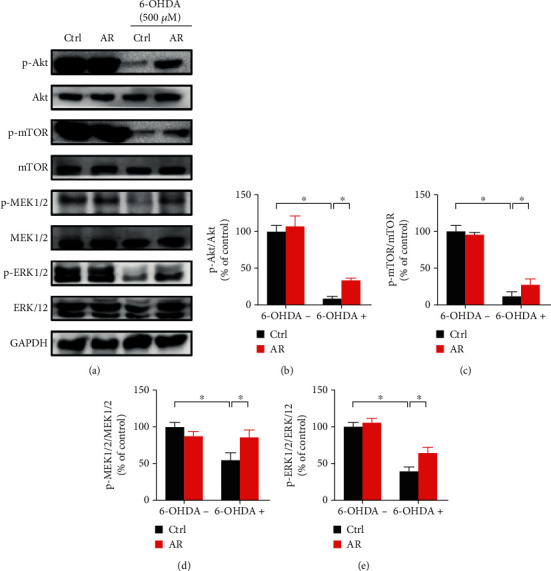
AR extract restores 6-OHDA-induced downregulation of the Akt/mTOR and MEK/ERK signaling pathway in PC12 cells. (a) PC12 cells were pretreated with 1 mg/mL AR extract or vehicle for 2 h and then treated with or without 500 *μ*M 6-OHDA for 24 h. Protein expression levels of p-Akt, Akt, p-mTOR, mTOR, p-MEK1/2, MEK1/2, p-ERK1/2, and ERK1/2 in PC12 cells were examined by Western blot analysis. (b–e) Quantitative analysis of protein expression levels. Data are presented as a percentage of control group values (mean ± SD of three independent experiments). ^∗^*p* < 0.05 indicates a statistically significant difference.

Akt/mTOR and MEK/ERK signaling pathways play key roles not only in the stimulation of neuron proliferation but also in the regulation of neuron apoptosis. Therefore, we studied the effects of AR extract on the changes in Akt/mTOR and MEK/ERK signaling pathways in PC12 cells. 500 *μ*M 6-OHDA decreased the expression of phospho-Akt, phospho-mTOR, phospho-MEK1/2, and phospho-ERK1/2 in PC12 cells by 92%, 88%, 45%, and 60%, respectively, whereas the total amounts of Akt, mTOR, MEK1/2, and ERK1/2 were not affected (Figure 8). With the pretreatment of PC12 cells with AR extract, the 6-OHDA-induced decrease in expression of phospho-MEK1/2 was completely restored, whereas the expression of phospho-Akt, phospho-mTOR, and phospho-ERK1/2 was partly restored.

### 3.10. Involvement of the Akt/mTOR and MEK/ERK Signaling Pathway in Protective Effect of AR against 6-OHDA-Induced Cell Injury in PC12 Cells

**Figure 9:**
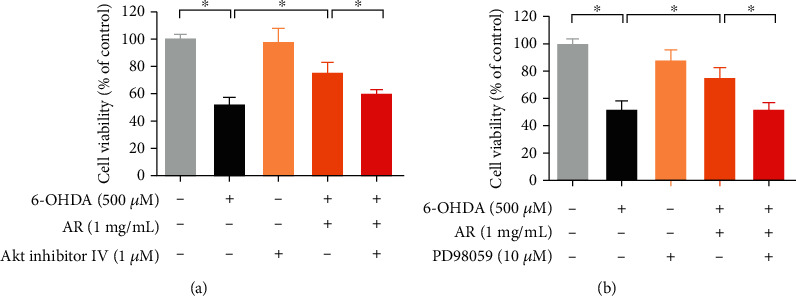
Akt inhibitor and MEK inhibitor abolish the neuroprotective effects of AR in 6-OHDA-treated PC12 cells. PC12 cells were pretreated with (a) 1 *μ*M AKT inhibitor IV or (b) 10 *μ*M MEK inhibitor (PD98059) for 30 min prior to incubation with 1 mg/mL AR for another 2 h, and then, the cells were exposed to 500 *μ*M 6-OHDA for 24 h to induce cell damage. Then, cell viability was examined with MTT assays. Data are presented as a percentage of control group values (mean ± SD of three independent experiments). ^∗^*p* < 0.05 indicates a statistically significant difference.

To further investigate the role of Akt/mTOR and MEK/ERK signals in protective effect of AR against 6-OHDA-induced cell injury in PC12 cells, Akt inhibitor IV and MEK inhibitor (PD98059) were applied to observe the neuroprotective effect of AR. As shown in Figure 9, 1 *μ*M Akt inhibitor had no effect on the cell viability of PC12 cells whereas 10 *μ*M MEK inhibitor (PD98059) slightly decreased the cell viability. Notably, both Akt inhibitor (Figure 9(a)) and MEK inhibitor (Figure 9(b)) abolished the protective effect of AR in PC12 cells. In 6-OHDA-induced cell injury model, the increase of cell viability induced by AR was significantly decreased from 75% to 58% and 52% in the presence of Akt inhibitor IV and PD98059, respectively.

## 4. Discussion

Oxidative stress is widely believed to be involved in the pathogenesis of many age-related diseases, such as neurodegenerative diseases, cardiovascular diseases, and cancer. Some antioxidants have been demonstrated to be effective in the prevention or treatment of oxidative stress-related diseases [16]. For instance, N-acetylcysteine can be used for the treatment of chronic obstructive pulmonary disease, in which oxidative stress is closely associated with its pathology and complications [17]. Vitamins are a class of essential micronutrients showing abundant antioxidant effects in humans. Their health-protective and therapeutic potential has been extensively studied. For instance, several large observational studies involving more than 100,000 participants have suggested that higher intake of vitamins significantly decreases the risk of coronary artery disease [18, 19]. Moreover, a community-based study in Rotterdam indicated that a high intake of dietary vitamin E may decrease the occurrence of Parkinson's disease in the population between 55 and 95 years of age [20].

*Ganoderma lucidum*, also known as “Lingzhi” in Chinese, is a well-known and popular edible and medicinal mushroom in Asia. In traditional Chinese medicine, it is used to promote health and longevity [21]. Numerous studies have demonstrated that *Ganoderma lucidum* exerts significant beneficial effects in neurodegeneration, diabetes mellitus, cardiovascular diseases, and tumor development [22]. These promising pharmacological activities of *Ganoderma lucidum* are at least partly attributed to its remarkable antioxidant and free radical scavenging activity. For instance, *Ganoderma lucidum* extract ameliorates MPTP-induced parkinsonism and protects dopaminergic neurons from oxidative stress via regulating mitochondrial function, autophagy, and apoptosis [23]. In a rat model, preadministration of *Ganoderma lucidum* was found to prevent hippocampus neurons from mitochondrial dysfunction and apoptosis by alleviating oxidative stress [24]. These findings suggest that other dietary mushrooms in the *Ganodermataceae* family may also exhibit potential effects in the prevention or treatment of oxidative stress-related diseases. AR is a species in the *Ganodermataceae* family whose pharmacological effects have rarely been explored. There were only two studies related to the chemical constituents of AR. These studies reported that ethanolic extract of AR contained phenolic compounds [13, 25], but there was no information about polysaccharides and triterpenes, which are well-known active ingredients in *Ganoderma lucidum*. Moreover, the major chemical contents of aqueous extract of AR have never been explored. This missing information is important because water decoction is the traditional method of extraction.

In the present study, the results of chemical assays demonstrated that the aqueous extract of AR contained phenolic compounds, polysaccharides, and triterpenes. The contents of total polysaccharides and triterpenes of AR extract were lower than that of aqueous extract of *Ganoderma lucidum* [26, 27]. Interestingly, the contents of total phenolic compounds of AR extract could reach the level of 5.52 mg GAE/g, which is unexpectedly much higher than that of aqueous extract of *Ganoderma lucidum* reported in other studies [28]. Indeed, numerous studies have shown that phenolic contents are highly correlated to antioxidant activities [29]. Therefore, we sought to investigate whether AR possess potential antioxidant activity, which may be useful in neuroprotection. At the beginning of the present study, we clearly demonstrated that the aqueous extract of AR had significant antioxidative activity in DPPH and TAC assays. In good agreement with the higher phenolic content of AR, the antioxidant capacity of AR extract was also stronger than that reported for aqueous extract of *Ganoderma lucidum* [26]. The antioxidant effect of AR extract was further examined in an *in vitro* model of Parkinson's disease, with PC12 cells. These cells are derived from the pheochromocytoma of the rat adrenal medulla, and they exhibit similar characteristics to those of neurons, owing to their common embryonic origin from the neural crest [12, 30]. PC12 cells have been widely adopted as an *in vitro* model to study neuronal development and neurological disease.

**Figure 10:**
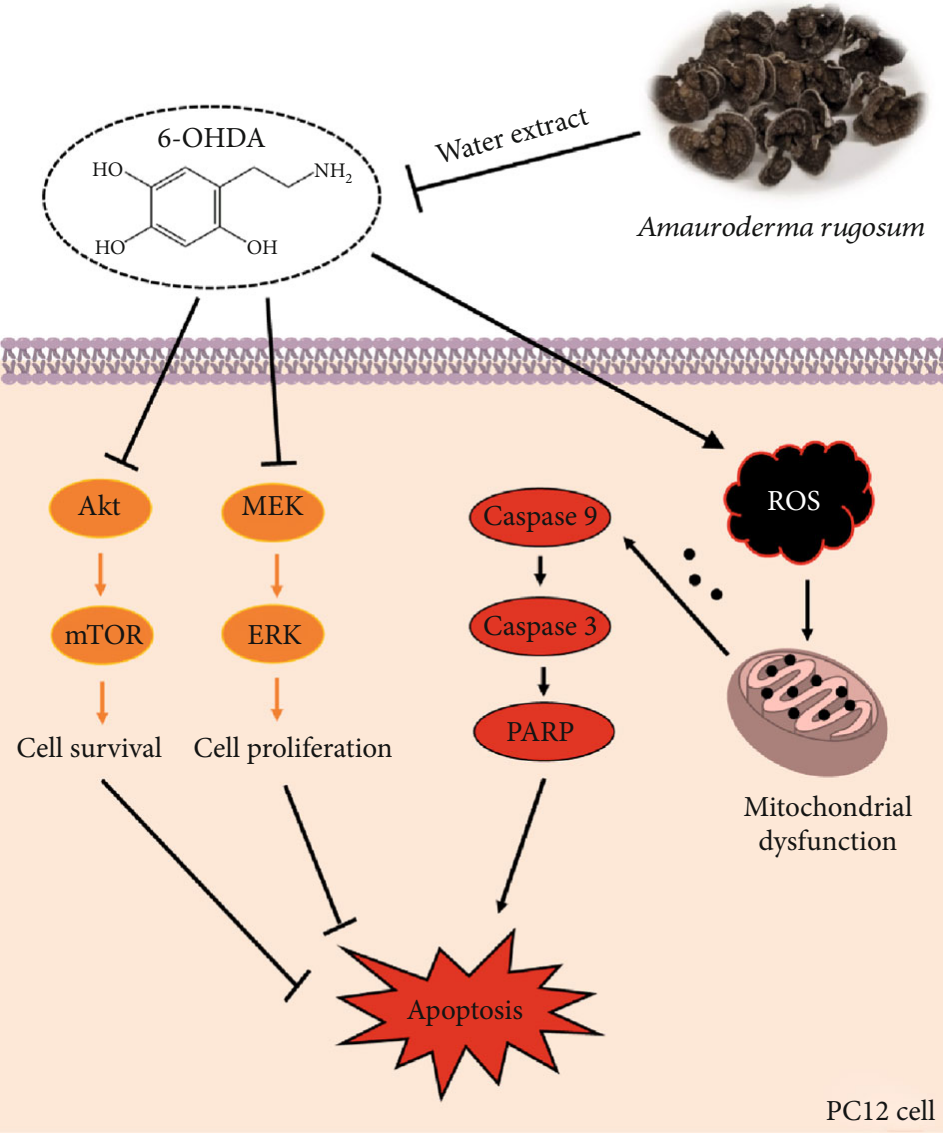
A schematic diagram illustrated the neuroprotective mechanisms of AR in PC12 cells.

In the present study, 6-OHDA was used to induce neurotoxicity in PC12 cells. In line with findings from other studies [11, 30], our results indicated that 6-OHDA significantly induced ROS generation, loss of mitochondrial membrane potential, and apoptosis in PC12 cells (Figure 10). Although PC12 cells share many similarities with neurons, they still cannot be regarded as true neurons [31]. Therefore, we used NGF-differentiated PC12 cells to compare the neuroprotective effects of AR with nondifferentiated PC12 cells. The results showed that AR could protect against 6-OHDA-induced neurotoxicity in both NGF-differentiated and nondifferentiated PC12 cells, which suggests the potential activity of AR in protecting neurons. Therefore, PC12 cells are a feasible and reliable *in vitro* model for studying the antioxidant and neuroprotective effects of AR extract.

Oxidative stress is considered as one of the major mechanisms contributing to dopaminergic neuron death in Parkinson's disease. Apart from being an important source of ROS production, mitochondria are also susceptible to oxidative stress. Increased oxidative stress can lead to mitochondrial dysfunction, which in turn triggers the further generation of ROS. Therefore, a vicious circle may form, in which oxidative stress and mitochondrial dysfunction feed each other forward [32]. The increased ROS production is involved in the reduction of mitochondrial ATP production, resulting in oxidative phosphorylation deficiency and then mitochondrial respiratory dysfunction [33]. 6-OHDA impairs oxidative phosphorylation and mitochondrial respiration through the direct suppression of mitochondrial complexes I and IV activity [1, 34]. Consistent with previous reports, we found that 6-OHDA were able to induce massive ROS generation and impair mitochondrial respiration severely in PC12 cells. AR extract could exert protective effects on PC12 cells by removing 6-OHDA-induced ROS generation and attenuating 6-OHDA-induced mitochondrial respiratory dysfunction, which revealed its promising neuroprotective activity.

In addition, emerging evidence has indicated that the accumulation of ROS causes mitochondrial damage and induces apoptosis via the upregulation of proapoptotic protein expression (e.g., caspase 3/7/9 and PARP), resulting in the loss of dopaminergic neurons in Parkinson's disease. The expression of cleaved-caspase 3/8/9 in dopaminergic neurons is significantly higher in people with Parkinson's disease than in healthy individuals [35, 36]. Similar results have also been observed in preclinical studies using *in vitro* and *in vivo* models [37, 38]. These findings have revealed the important role of apoptosis in the pathogenesis of Parkinson's disease. Drugs with antioxidant and antiapoptotic effects may provide an attractive way to protect neurons from cell death and slow the progression of neurodegeneration [39]. In this study, we found that 6-OHDA upregulated the expressions of proapoptotic proteins, such as cleaved-caspase 9, caspase 3, and PARP, in PC12 cells, but this effect was significantly decreased by AR extract. This experimental result suggests that the neuroprotective action of AR extract may be attributed to its inhibitory effects on proapoptotic protein expression.

Beyond apoptotic proteins, previous studies have reported that Akt/mTOR- and MEK/ERK-dependent signaling pathways regulate cell survival and proliferation in different types of cells, including endothelial cells, cancer cells, and neuronal cells [40–44]. Disruption of the molecular signals in Akt/mTOR- and MEK/ERK-dependent pathways may severely affect the development of Parkinson's disease. For instance, the activity of Akt and mTOR is significantly lower in the neurons of patients with Parkinson's disease compared with unaffected individuals. In postmortem dopaminergic neurons, the protein levels of phosphorylated Akt and mTOR are severely depleted, leading to neuron degeneration [45]. In addition, data from *in vivo* animal studies and *in vitro* neuronal and neuroepithelial cell studies have demonstrated that MEK and ERK promote neuron cell survival and proliferation by antagonizing cell death and apoptosis [46, 47]. Hence, Akt/mTOR- and MEK/ERK-dependent signaling pathways may be potential therapeutic targets for treating neurodegenerative diseases. In the present study, 6-OHDA strongly abolished the protein expressions of phospho-Akt, mTOR, MEK, and ERK in PC12 cells, suggesting that the activities of those signaling pathways might be greatly reduced. Interestingly, the effects of 6-OHDA on the expressions of these signaling molecules were completely or partly restored by AR extract, revealing that the underlying neuroprotective mechanisms of AR extract may involve the regulation of Akt/mTOR and MEK/ERK signaling transduction. Next, we used Akt inhibitor IV and MEK inhibitor (PD98059) to further investigate the role of Akt/mTOR and MEK/ERK signals in protective effect of AR against 6-OHDA-induced cell injury in PC12 cells. Notably, both Akt inhibitor IV and PD98059 abolished the protective effect of AR in PC12 cells, which suggests that the neuroprotective effects of AR in PC12 cells are possibly through the upregulation of Akt/mTOR and MEK/ERK signaling pathways.

In conclusion, this study has demonstrated that AR can protect PC12 cells from 6-OHDA-induced oxidative stress, mitochondrial dysfunction, and apoptosis. In addition to the direct ROS scavenging effect, AR extract may downregulate proapoptotic proteins and upregulate the Akt/mTOR- and MEK/ERK-dependent signaling pathways. This is the first study reporting the neuroprotective effect of AR. Our findings provide useful information for future investigation of the potential application of AR or its active ingredients in the prevention and treatment of oxidative stress-related neurodegenerative diseases such as Parkinson's disease.

## Data Availability

The data used to support the findings of this study are available from the corresponding author upon request.
